# Time and Location: Physical Activity Trends in Parents and Children in a Rural Region During COVID-19

**DOI:** 10.13023/jah.0701.01

**Published:** 2025-05-01

**Authors:** Brooke C. Towner, Robert Broce, Rebecca A. Battista

**Affiliations:** Appalachian State University; Appalachian State University; Appalachian State University

**Keywords:** Appalachia, Children, COVID-19, Family, Location, Physical Activity

## Abstract

**Introduction:**

COVID-19 restrictions altered the structure of the school and workday for families with children and led to changes in active transportation. Continued restrictions impacted access to many community locations and led to changes in physical activity (PA) behavior.

**Purpose:**

The objectives of the study were to describe the changes in the 1) amount of PA time per day, 2) frequency of PA, and 3) sitting time. Information about how PA changed for parents and children during the COVID-19 pandemic will have potential implications for education, public health, and recreation management.

**Methods:**

This cross-sectional study enlisted participants via an online survey to evaluate their and their children’s perceived PA behaviors before and during COVID-19. The study focused on parents in a rural Appalachian region within a Southeastern state, recruited through convenience sampling.

**Results:**

About one-third of parents reported an increase in time (37%) and frequency (33%) while two-thirds either stayed the same or decreased. Parents' time spent sitting increased in over half of the sample (50.8%). Parent perceptions of their children's changes in PA indicate that 49.2% reported lower recreational (i.e., free/leisure) PA, 13.7% reported less time participating in PA overall, and 43.8% noted a decrease in the number of days their child was physically active per week.

**Implications:**

Findings from this study show that trends in PA for rural families shifted during COVID-19 restrictions. Despite these restrictions, some parents demonstrated resilience by maintaining or increasing their PA levels. This highlights the need to further explore factors that support PA behaviors during periods of limited access to structured PA settings.

## INTRODUCTION

In the spring of 2020, the World Health Organization declared the SARS-Cov-2 virus, COVID-19, a global pandemic. To prevent the spread of COVID-19, governments implemented restrictions on human physical interactions (e.g., wearing masks and social distancing) and recommended staying at home and/or sheltering in place.^1^ Restrictions altered the structure of the school and workday for families with children and led to changes in active transportation and daily commuting as parents worked from home and children attended school virtually.

Continued restrictions impacted access to many community locations and led to changes in physical activity (PA) behavior. Public schools and non-essential businesses (e.g., gyms and recreational facilities) were closed, access to public trails, parks, playgrounds, and other recreational spaces were limited, and youth and college sports were canceled.^2^,^3^ Even in September 2020, outdoor playgrounds, fitness facilities, and facilities associated with competitive sports were only operating at 30% capacity.^1^ These public health efforts helped prevent the spread of COVID-19, but also limited options for daily PA leaving families tasked with finding creative ways to stay physically active. Some adults increasingly used their homes/garages and neighborhoods,^2^,^4^ while others turned to parks and other outdoor spaces to engage in PA.^4^

Parents and children engaging in adequate amounts of PA is a major health priority. Regular PA combats co-morbidities (obesity, hypertension, diabetes, and heart conditions) and enhances immune function.^5^ Short-term changes in PA, including increases in sitting and other sedentary behavior during the COVID-19 pandemic, may become typical behaviors increasing the risk of obesity, diabetes, and cardiovascular disease in children. Parents have reported continuing noticeable differences in their children's PA levels since the pandemic.^6^ With already increased obesity rates in children, families and caregivers must realize the importance of staying physically active while public health restrictions are in effect and how essential it is to return to PA after restrictions are lifted.^6^ Understanding the impact of the pandemic on PA participation may also provide insight in other situations that may restrict access to PA opportunities or lead to working and learning remotely (e.g., natural disasters, inclement weather).

Adults in rural communities tend to engage in less PA than those in urban areas.^7^ Additionally, adults who live in more isolated rural areas are even less active.^8^ The Appalachian Region faces elevated rates of chronic disease, health disparities, and limited access to healthcare. Not surprisingly, 30% of adults in the Appalachian Region are physically inactive.^9^ Before COVID-19, a 2019 National Center for Health Statistics report found that only about half of adults met 2008 federal PA guidelines for aerobic activity based on leisure-time activity.^10^ Those who lived in smaller metropolitan statistical areas (MSAs) or more rural areas outside of MSAs were less likely to meet the standard than those in larger MSAs (large MSA 54.1%, small MSA 49.4%, no MSA 42.5%).^11^ The precise impact of COVID-19-related public health restrictions on families residing in rural communities, particularly in the Appalachian Region, remains largely unknown. Before COVID-19, rural families faced access-related challenges like unreliable internet, digital technologies, lack of safe places to engage in PA, and lack of space available for PA in the home, yard, or neighborhood.^12^ Access to resources, socioeconomic status, geography, and environment can contribute to a lack of PA.^13^ These influences may have become even more pronounced during COVID-19.

As the structure of the daily routine shifted to a more localized, remote setting during COVID-19, some families discovered flexibility in their schedules, affording them more opportunities for PA in the neighborhood or at home. Conversely, with school closures and remote work, other families faced limitations on time and opportunities to engage in alternative physical activities. Closures, cancellations, and limits on PA participation during the pandemic prompted health and fitness professionals to deliver more online offerings (i.e., live or recorded) and provide exercise routines for home-based PA with limited equipment.^14^

Due to public health restrictions and the remote nature of school and work during COVID-19, PA may be severely impacted for parents and children in rural communities. Therefore, this study aimed to explore perceptions of PA before COVID-19 and during COVID-19 (September – October 2020) for parents and children in the Appalachian Region of Western North Carolina. The objectives of the study were to describe the changes in the 1) amount of PA time per day, 2) frequency of PA, and 3) sitting time. Given the potential for chronic diseases and mental health issues related to reduced PA, information about how PA changed for parents and children during the COVID-19 pandemic will have potential implications for education, public health, and recreation management.

## METHODS

### Participants

This cross-sectional study enlisted participants via an online survey to evaluate their and their children’s perceived PA behaviors before and during COVID-19. The study focused on parents in a rural Appalachian region within a Southeastern state, recruited through convenience sampling. Inclusion criteria included being 18 years or older, residing within a three-county area, having at least one child living in the household, access to an electronic device for survey completion, and proficiency in English. Consent to participate in the survey was granted by selecting the “agree” option on the first page of the survey. The study procedures were approved by the Appalachian State University Institutional Review Board.

### Data Collection

Data collection occurred from August – September 2020 through Qualtrics XM Online Survey Software. The survey required approximately 20 – 30 minutes to complete. During data collection, participants were under Phase 2/2.5 COVID-19 restrictions,^1^ which included safety at home recommendations, face-covering mandates, and reduced capacity for indoor and outdoor recreation and fitness facilities.^1^ The survey included sections on changes in perceived PA behaviors before and during COVID-19, focusing on time, frequency, and location. Participants could optionally provide their email to receive one of 150 $25 gift cards.

### Measures

#### Physical Activity

Participants self-reported perceived PA by location for themselves and their children.^6^,^15^,^16^ A 5-point Likert scale assessed perceived changes in both PA time and sitting time, ranging from “much lower” (1) to “much higher” (5). Participants reported PA participation before and during COVID-19 in five locations, while school was an additional location reported for their children. Frequency of PA participation was collected using a 3-point Likert scale, ranging from “frequently” (1) to “never” (3). Participants retrospectively reported weekly minutes of PA before and during COVID-19 for themselves. Weekly minutes of PA (time) at each location were reported using a 3-point Likert scale, ranging from “less than 30 minutes” (1) to “greater than 60 minutes” (3). For children, parents reported the frequency of their participation in PA at various locations before and during COVID-19.

#### Daily Living and Demographics

Standard questions from national surveillance systems (e.g., the National Health and Nutrition Examination Survey, NHANES) collected data on gender, age, race, education, income, relationship status, and employment (working — job site, home, or both/not working). Information about the household included the number of people and the presence of children under 18 years old, along with their age range.

### Statistical Analysis

Descriptive statistics, including frequencies, means, and standard deviations, were employed for demographic data and changes in perceived PA among parents and their children. IBM SPSS Statistics 28 was used for analysis. Categorical outcome variables were analyzed, including perceived PA time and frequency and sitting time. Changes in perceived PA time before and during COVID-19 were determined using Wilcoxon Signed-rank tests (p<0.05). Effect sizes (r) were calculated using the formula r = Z / √N and interpreted based on Cohen’s criteria: small (0.10–0.29), medium (0.30–0.49), and large (≥ 0.50).

## RESULTS

A total of 337 respondents completed the survey, and only 300 reported having children under 18 years living in the home. For the current analysis, 131 respondents were removed for missing data (under 50% of the survey completed), resulting in a final sample (n) of 169 participants with children in the home. Table 1 shows the descriptive statistics for the demographic characteristics of the sample. Most parents in the sample were female (87%), identified as white (96%), working (87%), reporting an annual household income between $90,000 to $149,999 (37%), and having children in the home between the ages of 12 – 17 years (95%).

### Parent Physical Activity

When parents compared their PA before and during COVID-19, about one-third reported an increase in time (37%) and frequency (33%) while two-thirds either stayed the same or decreased **(**Table 2). Parents' time spent sitting increased in over half of the sample (50.8%). A Wilcoxon Signed Rank Test showed that the median time spent in PA **(**Table 3) at several locations significantly differed during COVID-19. Self-reported daily PA time spent on home-based activities during COVID-19 (M = 2.08, SD = 0.77) was significantly higher than before COVID-19 (M = 1.81, SD = 0.79), Z = −3.65, p < .001, r = −.45 . In contrast, PA time at fitness facilities was significantly lower during COVID-19 (M = 1.39, SD = 0.79) compared to before (M = 2.19, SD = 0.74), Z = −3.91, p < .001, r = .94. Participation in recreational sports and intramurals was significantly lower during COVID-19 (M = 1.56, SD = 0.80) than before (M = 2.27, SD = 0.82), Z = − 4.33, p < .001, r = .96.

Parent perceptions of their children's changes in PA indicate that 49.2% reported lower recreational (i.e., free/leisure) PA, 13.7% reported less time participating in PA overall, and 43.8% noted a decrease in the number of days their child was physically active per week **(**Table 2). Similar to parents, the Wilcoxon Signed Rank Test showed that the frequency of PA for children **(**Table 4) during COVID-19 significantly differed across several locations. PA frequency in home-based activity increased during COVID (M = 1.57, SD = 0.64), Z = −3.94, *p* < .001, r = .50 (medium effect) compared to before COVID (M = 1.78, SD = 0.59). The frequency of recreational sports and intramurals significantly decreased during COVID (M = 2.63, SD = 0.63), Z = 9.10, *p* < .001, r = −.99 compared to before COVID (M = 1.69, SD = 0.85). Fitness facility frequency significantly decreased during COVID (M = 2.88, SD = 0.41), Z = 6.28, *p* < .001, r = .86 compared to before COVID (M = 2.40, SD = 0.79). The frequency of parks and trail use for PA significantly decreased during COVID (M = 1.84, SD = 0.72), Z = 2.73, *p* = .006, r = .31) compared to before COVID (M = 1.66, SD = 0.59). Only 13.0% of parents reported their children engaged in school-based PA (physical education, recess) for at least 60 minutes during COVID-19. No median difference in PA time was found for schools or neighborhoods.

## DISCUSSION

This project explored trends in rural parent and child PA during the COVID-19 pandemic. Data were collected during the Fall of 2020, amid Phase 2/2.5 COVID-19^1^, revealing significant shifts in PA behavior. While parents adapted by finding ways to maintain or increase their PA, children experienced declines, raising concerns about long-term health implications in a rural setting. Parents reported their children engaged in less PA, increased screen time, and adopted more sedentary behaviors. The location PA shifted with more activities occurring at home and decreased participation in organized sports, physical education, and community recreation. Although public health restrictions gradually eased, temporary behavioral changes, such as increased sitting, could continue, increasing the risk of chronic health conditions.

Contrary to studies reporting a 20–50% decline in adult PA during the early stages of the pandemic,^17^,^18^ our data, collected as COVID-19 restrictions were easing, indicate that parents maintained or increased their PA levels. This may be attributed to greater flexibility in scheduling, shifting work environments, and an increased focus on personal health. However, despite maintaining PA, over 50% of parents reported an increase in sitting time, aligning with national trends showing that sedentary behaviors increased during the pandemic.^17^,^19^ The transition to remote or hybrid work likely contributed to this rise, emphasizing the need for strategies addressing sedentary behavior and its associated health risks.

The pandemic also influenced where parents engaged in PA. Many shifted from fitness facilities and recreational sports as indicated by a large effect (r = .94 and r = .96, respectively) to home-based PA, outdoor trails, and neighborhood spaces. These large effect sizes suggest that the disruptions to these structured activities were not minor adjustments, but rather substantial behavioral shifts. Similar findings suggest previous fitness facility users adapted by leveraging virtual fitness programs, utilizing parks and trails, and exercising at home.^20^ While this demonstrates resilience, new challenges emerged, including relying on online resources, limited access to fitness equipment, and the overcrowding of local parks and trails.

Unlike parents, nearly 50% of children experienced decreased PA, coupled with increased sedentary behavior and screen time. This may have resulted from a lack of school-based PA, safety concerns, or parents prioritizing their own PA due to new work demands. Also, over 61% of children had increased sitting time. As schools transitioned to online instruction, children experienced prolonged screen time, reducing their engagement in active play. Previous research has suggested that children’s sedentary time increases with age, but the pandemic may have accelerated this trend.^21^ Without intentional interventions, these behaviors could become lifestyle habits, leading to negative health outcomes.^22^

Parents also reported that their child's PA time varied depending on location. Participation in PA time at parks and trails and recreational sports declined, reinforcing that public health restrictions limited access to organized and structured PA opportunities. Our results align with others^6^ suggesting that home became the primary location for children’s PA, yet this did not compensate for the lost opportunities in school and community settings. The large effect sizes we found for declines in structured PA settings (recreational sports, fitness centers) indicate that these losses were substantial. Additionally, many children engaged in less school-based PA, with only 13% achieving 60 minutes of PA during school hours, highlighting the need to reintegrate structured PA opportunities in schools, recreation programs, and community spaces to help children reach pre-pandemic PA levels.

### Implications and Recommendations

While considering the influence of COVID-19 on family PA, our study underscores the capacity of parents to maintain activity levels while highlighting children's increased trend of inactivity and sedentary behavior. While directly measuring PA would have been ideal, COVID-19 restrictions made this not feasible. Instead, we used self-report (i.e., parents' perceptions) to assess changes in PA. Parents' perceptions of their children's PA are important, as they play a key role in shaping PA habits in young children.^23^ However, it has been reported that parents often overestimate their children's PA, particularly when their children are less active.^24^ Additionally, this overestimation may stem from much of their children’s PA occurring at school, where parents have limited visibility. In the case of remote learning periods, such as during COVID-19, parents may have greater opportunities to observe and promote PA.^24^ COVID-19 prompted parents to seek new ways to keep their children active,^25^ a finding supported by our data. In contrast, with respect to the Appalachian Region, an association between parents’ and children’s sedentary behavior has been reported.^26^ Thus, when the home becomes a workplace and school, which may occur frequently in rural areas, it is important to understand how an environment influences behavior. These insights can inform different health sectors such as physical education, public health, and recreation management in efforts to promote PA among rural families.

### Physical Education

Physical education plays a critical role in ensuring that PA remains an integral part of students’ daily routines, even when traditional in-person instruction is disrupted. The shift to virtual learning during COVID-19 highlighted the need for structured, accessible, and engaging online physical education opportunities. Schools should integrate regular movement breaks into virtual and hybrid learning models and adopt policies and strategies that promote PA throughout the school day. Additionally, educators can leverage digital platforms to deliver interactive PE lessons, provide asynchronous activity challenges, and encourage families to participate in home-based activities. Schools must also advocate for outdoor and alternative PA settings, ensuring that students remain active regardless of the circumstances.

### Public Health

Public health agencies are essential in promoting accessible and sustainable PA options for families, especially in times of crisis or disaster. During the pandemic, public health restrictions limited access to recreational facilities, reinforcing the importance of home-based and outdoor PA options. Future public health initiatives should focus on increasing awareness of home- and community-based activity resources, providing guidelines for safe outdoor recreation, and expanding infrastructure that supports PA in various settings. Public health campaigns should also emphasize reducing sedentary behaviors, particularly among children, by promoting active family routines and neighborhood outdoor activities, sharing strategies to reduce screen time, and developing policies that integrate PA into daily life. Strengthening collaborations between public health, education, and recreation sectors can ensure that families have the knowledge and resources to maintain active lifestyles, even when typical options are unavailable.

### Recreation Management

The recreation management sector should continue to adapt and innovate to provide flexible and accessible PA opportunities for rural families. COVID-19 emphasized the need for recreation professionals to offer alternative programming, such as virtual classes, outdoor exercise options, and modified recreational sport leagues. Expanding the availability of adaptable PA programs can ensure smooth transitions between offerings and continued family engagement in PA. Additionally, recreation facilities and organizations should advocate for policies that promote access to parks, trails, and green spaces while considering barriers such as transportation, finances, and safety. By diversifying PA offerings, the recreation sector can support rural families in maintaining long-term active habits, regardless of external disruptions.

### Limitations

This study has several limitations. Firstly, participants were predominantly homogenous, drawn from a single rural community. The limited diversity in this sample is reflective of the broader demographics of rural Western North Carolina. Similar studies have reported comparable participant characteristics, including a high percentage of females,^3^,^6^ non-Hispanic individuals, and those with higher levels of education.^6^ According to the 2023 American Community Survey, the surveyed region's racial composition is 83.7% white, 3.9% black or African American, and 6.4% Hispanic or Latino.^27^ This lack of racial diversity presents a potential limitation in generalizing the findings to more diverse populations. Additionally, our sample had a high number of participants reporting income between $90,000–$149,999, with the median household income for families in the area being $87,273. Our sample had a high proportion of participants with postsecondary degrees compared to the national average. In 2019, about 35% of Americans had obtained some form of post-secondary education,^28^ whereas 82.6% of our participants reported holding a post-secondary degree. To enhance the external validity, future research should extend the survey to diverse rural communities. The use of a convenience sample from local listservs also limits the generalizability of the results despite efforts to incentivize participation for a more diverse sample.

Data collection relied on self-reported PA behavior and recalled behaviors, introducing potential biases. Moreover, while the questions used for PA and sedentary behavior have been published in other studies,^15^,^16^ their reliability and validity have not been assessed. While such methods are common, they may influence accuracy.^6^,^28^ Additionally, the survey coincided with the start of the school year, August – September 2020, a period of adjustment for families to online learning, potentially affecting responses.

Another limitation is grouping children by age range rather than specific ages, as varying activity levels and engagement patterns exist among younger children (three- to five-year-olds). Lastly, the use of the Wilcoxon Signed Rank Test, though standard, may be limited when data points are identical, potentially diluting the test's effect. Despite these limitations, our results suggest a meaningful change in PA based on location.

## CONCLUSION

Findings from this study show that trends in PA for rural families shifted during COVID-19 restrictions. Despite these restrictions, some parents demonstrated resilience by maintaining or increasing their PA levels. This highlights the need to further explore factors that support PA behaviors during periods of limited access to structured PA settings.

In contrast, children experienced greater declines in PA during COVID-19 as schools were closed and opportunities for recreational sports were canceled or restricted. This shift underscores the need for accessible, home or neighborhood-based programs that do not rely on travel, facilities, or structured participation, especially in rural areas where children experience transportation barriers and limited access to PA resources.

Results also reveal a notable shift with the home becoming the primary setting for PA for both parents and children. As public health restrictions eased, many parents continued home-based PA routines, suggesting potential long-term behavior changes. Moving forward, physical education programs must integrate meaningful at-home or out-of-school PA options to ensure rural students stay engaged. Recreational professionals in rural communities should also develop strategies to support PA during future disruptions. Future research should examine the specific types of home-based PA and factors influencing engagement and enjoyment, particularly in rural areas.

**SUMMARY BOX**

**What is already known about this topic?**

Previous research has indicated that the COVID-19 pandemic significantly disrupted daily routines and access to physical activity (PA) resources, leading to increased sedentary behavior and decreased PA, particularly among children. National trends showed a decline in PA levels among adults during the early stages of the pandemic, and studies also highlighted barriers to PA in rural areas due to limited access to facilities, transportation, and the internet. Rural populations, especially in the Appalachian Region, had already faced higher rates of inactivity and chronic health issues prior to the pandemic.

**What is added by this report?**

This study provides insights by specifically examining changes in parent and child physical activity (PA) behaviors during COVID-19 within a rural Appalachian community. It highlights that, contrary to national trends, many parents in this region maintained or increased their PA despite public health restrictions, primarily by shifting activities to the home environment. However, children saw a significant decline in PA, mainly due to the loss of school-based and recreational opportunities. The report also emphasizes the home as a key location for PA during disruptions and suggests that these behavior changes may persist even after restrictions have eased.

**What are the implications for future research?**

Future research should (1) investigate the specific types of home-based physical activities that parents and children participate in, (2) examine the role of parental influence and home environments in shaping children's physical activity behaviors during both crisis and non-crisis periods, and (3) expand studies to include a wider range of rural populations to help strengthen the applicability of findings and inform public health and education strategies to promote physical activity. Additionally, longitudinal studies could evaluate the lasting impact of increased sedentary behavior and decreased structured physical activity, especially among children whose routines have been most disrupted.

## Supplementary Information



## Figures and Tables

**Table 1:** Basic Demographics of Parents Surveyed during August – September 2020

	N (%) or Mean ± SD
**Age (years)**	44.8 ± 7.27
**Gender**	
Males	23 (13%)
Females	146 (87%)
**Ethnicity**	
White	163 (96%)
Black or African American	0 (0%)
Indigenous American or Alaska Native	3 (2%)
Other	3 (2%)
**People in Household**	
1–3	57 (34%)
4–6	106 (63%)
>7	6 (3%)
**People in Household under 18**	
1	59 (37%)
2	62 (39%)
3	28 (18%)
>4	10 (6%)
**Age of People in Household under 18** ^*^	
0 – 5 years	36 (21%)
6 – 11 years	73 (43%)
12 – 17 years	161 (95%)
**Employment**	
Working at the job site	47 (28%)
Working from home	52 (31%)
Working from both (job site and home)	47 (28%)
Not working	21 (13%)
No response	2 (1%)
**Highest level of school completed**	
High school graduate	4 (2%)
Some college but no degree	21 (13%)
Associate degree	12 (7%)
Bachelor’s degree	61 (36%)
Graduate degree (Masters, Doctoral, Professional)	69 (41%)
Prefer not to respond	2 (1%)
**Annual household income from all sources**	
< $10,000	2 (1%)
$10,000 to 29,000	6 (4%)
$30,000 to 49,000	13 (8%)
$50,000 to 69,000	20 (12%)
$70,000 to 89,0000	32 (19%)
$90,000 to 149,000	63 (37%)
>$150,000	18 (10%)
Prefer not to respond	15 (9%)

NOTE:

Participants were asked to check each applicable age range.

**Table 2:** Self-Reported Changes in Perceived Physical Activity (Time and Frequency) and Sitting (Time) Among Parents and Children (n=169) During the COVID-19 Pandemic

	Adults	Children
**Time**	**N (%)**	**N (%)**
Much Lower	20 (11.8)	30 (17.8)
Somewhat Lower	33 (19.5)	55 (32.5)
About the same	54 (32.0)	28 (16.6)
Somewhat higher	50 (29.6)	34 (20.1)
Much higher	12 (7.1)	19 (11.2)
Not applicable		2 (1.2)
**Frequency (days per week)**	**N (%)**	**N (%)**
Much Lower	23 (13.6)	32 (18.9)
Somewhat Lower	30 (17.8)	42 (24.9)
About the same	59 (34.9)	47 (27.8)
Somewhat higher	41 (24.3)	30 (17.8)
Much Higher	15 (8.9)	15 (8.9)
Not Applicable	1 (0.6)	2 (1.2)
**Sitting Time**	**N (%)**	**N (%)**
Much Lower	5 (3.0)	5 (3.0)
Somewhat Lower	23 (13.6)	18 (10.7)
About the same	52 (30.8)	38 (22.5)
Somewhat higher	48 (28.4)	55 (32.5)
Much Higher	38 (22.5)	47 (27.8)
Not applicable	3 (1.8)	6 (3.6)

NOTE: Answers were reported using a Likert scale.

**Table 3:** Comparison of Self-Reported Time Spent Being Physically Active per Day at Various Locations Before and During COVID-19 for Parents and Children (n=169)

Location	Before Mean (SD)	During Mean (SD)	Z	P-value*	r†
**Parents**					
Parks/Trails	2.01 (0.76)	2.03 (0.82)	0.31	0.76	−0.04
Recreational sports/intramurals*	2.27 (0.82)	1.56 (0.80)	−4.33	<0.001	0.96
Neighborhoods	1.67 (0.71)	1.71 (0.73)	0.74	0.46	−0.10
Home-based activity*	1.81 (0.79)	2.08 (0.77)	−3.65	<0.001	−0.45
Fitness facilities*	2.19 (0.74)	1.39 (0.79)	−3.91	<0.001	0.94

NOTE: Means and SD values are based on a 3-point Likert Scale using the following for time spent physically active at each location: 1 = < 30 minutes 2 = 30–60 minutes 3 = > 60 minutes. Differences were calculated as During from Before.

* P<0.05, indicates a statistically significant change based on the Wilcoxon-signed rank test.

† The measure of effect size, *r*, is the rank biserial correlation.

**Table 4:** Comparison of Parent-Reported Frequency of Children Being Physically Active at Various Locations Before and During COVID-19 (n=169)

Location	Before Mean (SD)	During Mean (SD)	Z	P-value*	r†
**Children**					
Parks/Trails*	1.66 (0.59)	1.84 (0.72)	2.73	0.006	0.31
Recreational sports/intramurals*	1.69 (0.85)	2.63 (0.63)	9.10	<0.001	0.99
Neighborhoods	1.88 (0.60)	1.84 (0.69)	−0.66	0.51	0.09
Home-based activity*	1.78 (0.59)	1.57 (0.64)	−3.94	<0.001	0.50
Fitness facilities*	2.4 (0.79)	2.88 (0.41)	6.28	<0.001	0.86
School (physical education/recess)	2.19 (0.87)	2.32 (0.70)	1.71	0.09	0.19

NOTE:

Means and SD values are based on a 3-point Likert Scale using the following for time spent physically active at each location: 1 = frequently 2 = sometimes 3 = never. Differences were calculated as During from Before.

* P<0.05, indicates a statistically significant change based on the Wilcoxon-signed rank test.

† The measure of effect size, *r*, is the rank biserial correlation.
