# Supplementary material for: Time and Location: Physical Activity Trends in Parents and Children in a Rural Region During COVID-19
Source: J Appalach Health. 2025 May 1;7(1):1–21. doi: 10.13023/jah.0701.01 (PMC12111983; doi:10.13023/jah.0701.01)
Supplement: Supplementary file 1 [file 7.1.1_Towner_Additionalfile.pdf]

Time and Location: Understanding Physical Activity in Parents and Children During COVID-19

Rural communities tend to engage less in PA than their urban counterparts

Government restrictions impacted the accessibility of traditional means of PA

Phyiscal activity is major health priority

Online Survey of Parents' self-reporting Physical Activity Pre and During Pandemic

N =169

3 Rural Counties in Western NC

Adults (18+) with at least one child

PARENTS PA

Over 50% achieved 30 minutes daily at Parks/Trails and 64.1% did at home

CHILDRENS PA

Parents perceived that children's PA decreased

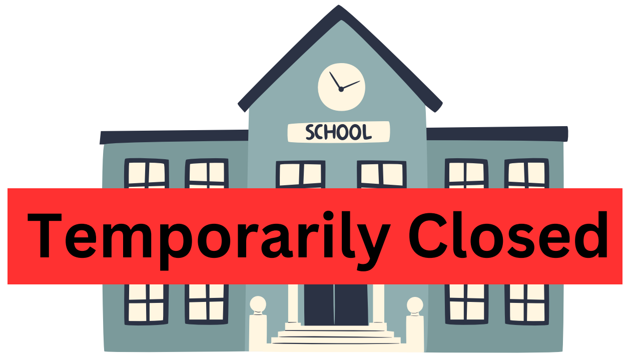

Summary:

Parents found ways to maintain or increase physical activity while children's PA suffered. The inability of children to maintain PA levels, given the restrictions based on locations, is concerning. Improving PA resources in or near the home is warranted.

Journal Information:
Website:
Doi:
